# Multiple Community Properties Drive Ecosystem Resistance and Resilience to Extreme Climate Events Across Mesic Grasslands

**DOI:** 10.1111/ele.70380

**Published:** 2026-04-07

**Authors:** Joshua A. Ajowele, Ashley L. Darst, Nameer R. Baker, Rachael R. Brenneman, Caitlin Broderick, Seraina L. Cappelli, Maowei Liang, Mary Linabury, Matthew A. Nieland, Maya Parker‐Smith, Smriti Pehim Limbu, Rosalie S. Terry, Moriah L. Young, Max Zaret, Marissa Zaricor

**Affiliations:** ^1^ Department of Biology University of North Carolina Greensboro Greensboro North Carolina USA; ^2^ Konza Prairie Biological Station Kansas State University Manhattan Kansas USA; ^3^ Department of Integrative Biology Michigan State University East Lansing Michigan USA; ^4^ W.K. Kellogg Biological Station Michigan State University Hickory Corners Michigan USA; ^5^ Ecology, Evolution, and Behavior Program Michigan State University East Lansing Michigan USA; ^6^ Department of Ecology, Evolution, and Behavior University of Minnesota St. Paul Minnesota USA; ^7^ Cedar Creek Ecosystem Science Reserve University of Minnesota East Bethel Minnesota USA; ^8^ School of Life Sciences, Global Drylands Center Arizona State University Tempe Arizona USA; ^9^ Graduate Degree Program in Ecology, Department of Biology Colorado State University Fort Collins Colorado USA; ^10^ Stockbridge School of Agriculture University of Massachusetts Amherst Massachusetts USA; ^11^ Denison University Granville Ohio USA; ^12^ Department of Environmental Studies Dartmouth College Hanover New Hampshire USA; ^13^ Division of Biology Kansas State University Manhattan Kansas USA; ^14^ Department of Plant Biology Michigan State University East Lansing Michigan USA

**Keywords:** aboveground biomass, biodiversity, climate change, context dependency, drought, ecosystem stability, nitrogen, nutrients, productivity, wet events

## Abstract

Ecosystem resistance and resilience to extreme climate events is impacted by community properties, including biodiversity. However, the relative importance of species richness, evenness and dominance is debated and is further modulated by global change factors such as nutrient addition. Using nearly 40 years of data from naturally‐assembled plant communities at three Long‐Term Ecological Research sites, we found that while species richness is important for resistance to extreme dry events, dominance is important for resistance to extreme wet events and evenness is important for resilience under ambient (unfertilized) conditions. Furthermore, nutrient addition alters resistance and resilience indirectly by reducing species richness and increasing dominance. Species richness and dominance are also directly reduced by extreme climate events, which may erode resistance and resilience to future events. Our results show that species richness, dominance and evenness shape ecosystem stability under climate extremes and that fertilization fundamentally modifies biodiversity–stability relationships in mesic grasslands.

## Graphical abstract

Using nearly 40 years of data from naturally‐assembled plant communities, we found that while richness is important for resistance to extreme dry events, dominance is important for resistance to extreme wet events and evenness is important for resilience. Furthermore, nutrient addition alters resistance and resilience indirectly by reducing richness and increasing dominance. Richness and dominance are also directly reduced by extreme climate events, which may erode stability to future events.

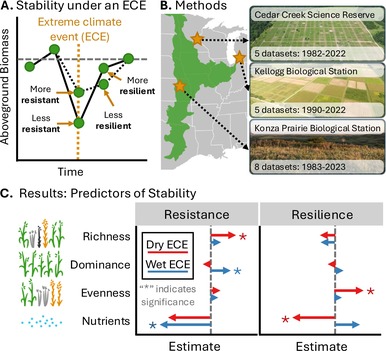

## 1 Introduction

Resistance and resilience as components of ecosystem stability underpin the capacity to maintain ecosystem functions like biomass production under more variable and extreme climate events (IPCC 2023; Wang, Isbell, et al. 2021). Resistance refers to an ecosystem's ability to maintain function during a disturbance (i.e., minimal change in response to disturbance), whereas resilience describes the recovery of the ecosystem function to a pre‐disturbed state following disturbance (Holling 1973; Isbell et al. 2015). These stability components can be influenced by biodiversity—particularly species richness, evenness and dominance—through differential species response to perturbation (Angeler and Allen 2016; Holling 1973; Loreau et al. 2021; Tilman et al. 2014). However, the relative importance of species richness, evenness and dominance in governing ecosystem resistance and resilience remains debated (Hoover et al. 2014; Lisner et al. 2024; Perez et al. 2024; Smith et al. 2020).

There is support for multiple community properties as key regulators of ecosystem stability. Some studies emphasize that greater species richness buffers ecosystems against disturbance by increasing the likelihood that some species will maintain biomass production during a disturbance event (Hossain et al. 2023; Isbell et al. 2015; Pfisterer and Schmid 2002; Tilman and Downing 1994). For example, under dry conditions, higher species richness may increase resistance via insurance effects (Tilman and Downing 1994; Yachi and Loreau 1999) by increasing the probability that drought tolerant species will maintain biomass production when others decline (Chen et al. 2023; Isbell et al. 2015; Tilman and Downing 1994; Xu et al. 2022). Others argue that dominant species primarily drive ecosystem functioning through their large contributions to biomass and resource use. This ‘mass‐ratio’ view of ecosystem functioning (Grime 1998) posits that community level processes may depend more on the performance and persistence of dominant species than overall biodiversity (Avolio, Forrestel, et al. 2019; Smith and Knapp 2003; Zhang et al. 2025). For example, in North American tallgrass prairies, C_4_ grasses possess traits that allow rapid acquisition of resources during wet conditions (O'Keefe and Nippert 2018) or under nutrient limiting conditions (Harpole and Tilman 2006) which influence community‐level biomass responses (Smith et al. 2020). Evenness may also play an important role in determining ecosystem stability (Wang, Ge, et al. 2021; Yan et al. 2021), as the resilience to extreme events may rely on how evenly biomass is distributed among species. Higher evenness promotes niche complementarity and asynchronous species dynamics in some systems, allowing species to differentially compensate over time and enhance recovery following disturbances (Orwin et al. 2014; Thibaut and Connolly 2013). However, the influence of richness, dominance and evenness on resistance and resilience are increasingly modified by global change drivers, including eutrophication and climate extremes (Davidson et al. 2025; Mohanbabu et al. 2025).

Among these global change drivers, nutrient pollution, largely caused by fossil fuel combustion and agricultural inputs (Galloway et al. 2004), is especially pervasive in grassland ecosystems. Nutrient addition can reduce plant diversity while increasing dominance of a few species or reordering dominant species (Avolio et al. 2014; Smith et al. 2020; but see Seabloom et al. 2021), potentially increasing mean productivity in the short term (Isbell et al. 2013) but undermining resilience over longer timescales (Bharath et al. 2020; Hautier et al. 2014). Yet, empirical results on the effects of nutrient addition on resistance and resilience remain mixed: some report increases (Carlsson et al. 2017), others report declines (Meng et al. 2021; Van Sundert et al. 2021) and still others find no significant (Chen et al. 2024) or divergent effects (Bharath et al. 2020; Xu et al. 2022). Furthermore, the type of climate extreme (wet or dry) likely mediates ecosystem responses. Indeed, most work has focused on drought limiting plant biomass production (Grossiord et al. 2014; Smith et al. 2024; Tilman and Downing 1994), yet extreme wet events are also projected to increase with climate change (IPCC 2023) and remain comparatively understudied (but see Hossain et al. 2022; Isbell et al. 2015). Many grasslands worldwide are water limited (DeMalach et al. 2017) such that above‐average wet years may enhance productivity (Knapp et al. 2001), but too wet of conditions could reduce production and reorganize plant communities (Grant et al. 2014; Hudson et al. 2022). Nutrient availability also interacts with climatic extremes, modifying productivity (Fay et al. 2025) and potentially altering the resistance and resilience of ecosystem functions (Bharath et al. 2020; Tilman and Downing 1994). Thus, investigating ecosystem resistance and resilience under different climate and nutrient alteration contexts may shed light on the underlying mechanisms of grassland stability.

Although numerous studies have advanced our understanding of biodiversity effects on stability, most rely on short‐term manipulations or are limited to single sites, specific experiments and specific climate events (Chen et al. 2024; Cusser et al. 2020; Tilman and Downing 1994), reducing their generalizability across broader ecological contexts. Other studies use long‐term biodiversity experiments to examine the effects of manipulated species richness on ecosystem stability (Hossain et al. 2022; Isbell et al. 2015), but other community properties such as dominance and evenness are more difficult to manipulate experimentally and may not represent naturally‐assembled communities. Therefore, there is a need for studies that combine multi‐decadal, multi‐site data from naturally‐assembled communities that have experienced multiple extreme dry and wet events. Here, we address this gap by analysing nearly 40 years of data from naturally‐assembled plant communities at three ecologically similar yet geographically distinct tallgrass prairie sites in the Midwestern United States. These Long‐Term Ecological Research (LTER) sites share many common species and experience similar climate variability, offering a rare opportunity to assess how multiple components of biodiversity jointly regulate the resistance or resilience of plant biomass under naturally occurring extreme climate events. We test how species richness, evenness and dominant species abundance drive resistance and resilience to extreme dry and wet events, and how nutrient addition modifies these relationships in three grasslands (summarized predictions in Table S1). In addition, we test if nutrient addition directly changes resistance and resilience, or indirectly changes resistance and resilience through its associated effects on plant community properties. Lastly, we assess how plant biomass and community properties respond during extreme events which could create feedbacks that further destabilize ecosystems as extreme climate events increase in frequency. This combination of long‐term, multi‐site data from naturally‐assembled grassland communities provides critical insight beyond grasslands with experimentally manipulated communities and enhances the ecological generality of our findings across the historic extent of tallgrass prairie in North America and other mesic grassland ecosystems.

## 2 Methods

### 2.1 Site Description

We compiled data from three Long‐Term Ecological Research (LTER) sites (Cedar Creek (CDR), Kellogg Biological Station (KBS) and Konza Prairie (KNZ)) with mesic grasslands that are located across the historical expanse of the tallgrass prairie. Spanning the Midwestern United States, these sites share a common suite of dominant C_4_ and C_3_ grass species, including *Andropogon gerardii* Vitman, *Schizachyrium scoparium* (Michx.) Nash, *Elymus repens* (L.) Gould, *Poa pratensis* L., *Sorghastrum nutans* (L.) Nash and *Panicum virgatum* L. (Eckberg et al. 2023; Risser 1981). We focused on these three sites because they combine ecological relevance, long‐term experimental infrastructure and comparability across climate, making them ideal for addressing questions about ecosystem resistance and resilience in mesic grasslands (Hudson et al. 2022). The sites span an 867 km longitudinal range, 184 mm years^−1^ mean annual precipitation (MAP) range and 6.2°C mean annual temperature (MAT) range (CDR 1982–2022: MAP = 776 mm years^−1^, MAT = 6.7°C; KBS 1990–2022: MAP = 960 mm years^−1^, MAT = 9.3°C; KNZ 1984–2023: MAP = 866.6 mm years^−1^, MAT = 12.9°C). CDR is located on sandy soils in East Bethel, Minnesota, USA (45.401°, −93.201°) and contains oak savanna, prairie, abandoned agriculture fields and forest (Hodson and Alexander 1985). KBS is located on sandy and silty clay loam soils in Hickory Corners, Michigan, USA (42.40°, −85.40°) and contains restored prairie, early successional habitat and agricultural fields (Crum and Collins 1995; Robinson et al. 2013). KNZ is located on silt loam and silty clay loam soils in Manhattan, Kansas, USA (39.093°, −96.575°) and contains remnant tallgrass prairie (Ransom et al. 1998).

### 2.2 Data Compilation

We screened all available data from the three LTER sites and compiled all datasets that contained at least five consecutive years of plant aboveground biomass and community composition data (Table S2). We only retained studies with naturally‐assembled grasslands, including studies that contained both control and treatment plots (e.g., nutrient addition). The included experiments at each site were subject to varying burning frequencies (annually–every 5 years) to maintain the grasslands. We excluded actively managed agricultural plots and plots in which plant diversity was actively manipulated via weeding. Further, we excluded plots with continuing disturbance (i.e., tilling), irrigation, grazing, insecticides and fungicides. Lastly, we excluded previous year dead litter from the plant biomass data.

We divided plots into two categories: no nutrient addition (‘control’) plots and nutrient‐addition plots. For nutrient‐addition plots, we included any nutrient addition with nitrogen (N, NP, NK, NPK). We excluded nutrient‐addition plots that did not apply nitrogen (such as potassium only) because all three sites are generally considered nitrogen limited (Avolio et al. 2014; Inouye et al. 1987; Waterton et al. 2022; Wilcots et al. 2025). While other nutrients are also important for primary production (Fay et al. 2015, 2025), we lacked the data to test for the effects of nutrients besides nitrogen. In total, 18 datasets across the three sites fit our criteria. Plant species names in the combined dataset were updated and matched with the ‘TNRS’ package and the underlying databases (Boyle et al. 2013, 2021; Govaerts 2023; Korotkova et al. 2021; The World Flora Online Consortium 2023). Harmonized data are available from the Environmental Data Initiative: https://doi.org/10.6073/pasta/433a9ec43bfca734604b8c874895c991 (Ajowele et al. 2026).

We used the Standardized Precipitation Evapotranspiration Index (SPEI) to quantify extreme wet and dry climate events. SPEI is a measure of climatic water balance and is the standardized difference between precipitation and potential evapotranspiration which can be calculated at different monthly timescales (Vicente‐Serrano et al. 2010). We downloaded SPEI‐3, 6, 9 and 12 for the month of August (i.e., August and the 2, 5, 8 and 11 months prior) at each of the sites from SPEIbase version 2.10 at the spatial resolution of 0.5 degrees using climate from 1901 to 2024 (Beguería et al. 2024). We selected the month of August since it represents the typical harvest month to quantify aboveground biomass across the three sites. To find which SPEI duration explained the natural variation in aboveground biomass best, we created linear mixed effects models using the ‘lme4’ package (Bates et al. 2015) with the natural‐logarithm+1 of aboveground biomass in control plots as a function of SPEI, an optional quadratic term and plot nested in site as random effects for each SPEI duration. We used the ‘bbmle’ (Bolker et al. 2023) and ‘MuMIn’ (Barton 2025) packages to compare AICc and marginal *R* ^2^ for the SPEI duration models. Comparisons between models with or without the quadratic term revealed the SPEI‐9 model had the lowest AICc (dAICc > 2) and the highest marginal *R* ^2^. Therefore, we used SPEI‐9 to define extreme climate events (Figure S1, Table S3). SPEI‐9 includes both the growing season and pre‐season, which may be important for recharging soil moisture prior to plant growth (Robinson et al. 2013). We defined an extreme event as occurring once every 10 years (SPEI threshold = ±1.28) (Isbell et al. 2015). In total, we identified 28 extreme events across our three sites: 10 extreme wet events and 2 extreme dry events at CDR, 9 extreme wet events and 1 extreme dry event at KBS and 3 extreme wet events and 3 extreme dry events at KNZ (Figure S2). Of these extreme events, two dry events and five wet events were simultaneously experienced in at least two sites.

### 2.3 Response Metrics

We used resistance and resilience as measures of ecosystem stability to extreme climate events, following the definitions in Isbell et al. (2015). Resistance (Ω) and resilience (Δ) are defined as

$$\Omega =\frac{\bar{Y_{n}}}{\left|Y_{e}-\bar{Y_{n}}\right|}$$

$$\Delta =\left|\frac{Y_{e}-\bar{Y_{n}}}{Y_{e+1}-\bar{Y_{n}}}\right|$$

in which $\bar{Y_{n}}$ is mean plant biomass during normal climate years, $Y_{e}$ is plant biomass during an extreme event and $Y_{e+1}$ is plant biomass the year after an extreme event.

To quantify plant community properties, we calculated species richness, evenness and dominance in each plot from relative abundance derived from percent cover or biomass data. We first removed non‐plant material (e.g., fungi, miscellaneous litter) from species composition data before calculating the diversity indices. Species richness was calculated as the number of unique plant species. Community evenness was calculated as *E*_var_ in the ‘codyn’ package (Avolio, Carroll, et al. 2019; Hallett et al. 2016), which is independent of species richness and is recommended as an evenness index (Smith and Wilson 1996). For dominance, we first identified the dominant species in each plot as the species that had the most years with the highest relative abundance in that plot. Recent work suggests long‐term dominant species contribute the most to functioning and positive biodiversity‐ecosystem functioning relationships, while short‐term dominants may not affect or even negatively affect functioning (Allan et al. 2025). Dominance was then selected as the relative abundance of that species in each year (Perez et al. 2024). This allows dominance to be more independent of species richness and evenness and allows us to account for the identity of the dominant species in each plot (Avolio, Forrestel, et al. 2019). The seven most common dominant species in our dataset are *Andropogon gerardii* , *Schizachyrium scoparium* , *Elymus repens* , *Poa pratensis* , *Solidago canadensis* L., *Sorghastrum nutans* and *Panicum virgatum*, which are commonly dominant in prairie communities (Eckberg et al. 2023; Risser 1981).

To assess the effects of extreme climate events on species richness, evenness, dominance and plant biomass, we used log response ratios (LRRs) to document the magnitude and direction of responses to an extreme climate event. We calculated the LRR as follows:

$$\mathrm{LRR}=\ln\left(\frac{Y_{e}}{\;\bar{Y_{n}}}\right)$$

in which $\bar{Y_{n}}$ is the mean community metric during normal climate years (SPEI‐9 < 0.67 and > −0.67) and $Y_{e}$ is the community metric during an extreme event (SPEI‐9 ≥ 1.28 and ≥ −1.28). We compiled all data using R statistical software (R Core Team 2025).

### 2.4 Statistical Analyses

We asked how species richness, evenness and dominance affected the resistance and resilience of aboveground biomass to extreme climate events. For these models, we used species richness, dominance and evenness from the year prior to the extreme event instead of mean values for these metrics (Perez et al. 2024). The prior year community property metrics best reflect impacts on resistance and resilience in the year of interest since our study did not manipulate plant community properties and thus they may vary interannually. For the resilience model, we dropped data points when there was an extreme event after an extreme event. We first constructed two linear mixed effects models with the natural logarithm of resistance or resilience as a function of species richness, dominance, evenness, event type (dry and wet), treatment (no nutrient addition and nutrient addition), the interactions between each community property and event type and interactions between each community property and treatment with plot nested in experiment nested in site and year as random intercepts (Equation S1; Bates et al. 2015). The inclusion of experiment in the random effect structure allowed us to account for differences associated with each experiment (e.g., fire frequency). Species richness, evenness and dominance measures were weakly correlated in our models (correlation < |0.38|), and thus we included them together in the analyses without collinearity concerns (VIF < 3). We consecutively dropped interaction terms from each model and performed model selection using log‐likelihood (Zuur et al. 2009). The model selected for resistance included the interaction between richness and event type, and the model selected for resilience included an interaction between richness and event type, an interaction between dominance and nutrient addition and an interaction between evenness and nutrient addition (Table S4, Figure S3). We then ran separate linear mixed effects models for the extreme dry and wet event scenarios using the best models selected for resistance and resilience, excluding the event type main effect (Equations S2 and S3). To test the robustness of the models, we performed leave‐one‐out sensitivity analyses by sequentially removing sites or years from the models (Figures S4 and S5). However, there were not enough data to conduct a sensitivity analysis for the resilience to extreme dry events model.

To test if nutrients act directly on resistance and resilience or indirectly through changes in plant community properties from the year prior to extreme climate events, we used structural equation models (SEM). First, we constructed a conceptual framework a priori based on hypothesized relationships (Table S1). We then created a multigroup structural equation model grouped by extreme event type (wet and dry) with plot nested in experiment as a random intercept using the ‘piecewiseSEM’ package (Lefcheck 2016). We used the ‘piecewiseSEM’ package to account for experiment‐specific responses in the random effects. All variables were centered and scaled prior to implementing the SEM. ‘PiecewiseSEM’ does not offer model fit indices that are accurate with datasets as large as ours. Therefore, the goodness‐of‐fit test in ‘piecewiseSEM’ is very likely to indicate poor model fit (Lefcheck 2016), as demonstrated by our multigroup ‘piecewiseSEM’ model (Fisher's C = 44.67, *p* < 0.0001, df = 6). We fitted the same model in the ‘lavaan’ package (Rosseel 2025) to (a) verify our results with a second statistical approach and (b) have additional information on how well our data fit a model containing the hypothesized paths. We accounted for sampling structure using ‘lavaan.survey’ (Oberski 2014). Refitting the model in ‘lavaan’ demonstrated good model fit with tests less sensitive to sample size (CFI = 0.96, SRMR = 0.04, IFI = 0.96) except for one (RMSEA = 0.10). Qualitatively, the ‘lavaan’ model gave the same results as the ‘piecewiseSEM’ model. We thus only report the results of the ‘piecewiseSEM’ model. To obtain *R* ^2^ and covariance values, additional ‘piecewiseSEM’ models were fitted separately for extreme wet and dry events.

To understand how the plant community was affected by extreme events, we used linear mixed effects models with the LRR of plot biomass, species richness, dominance or evenness as a function of the interaction between event type (dry and wet) and treatment (no nutrient addition and nutrient addition) with plot nested in experiment nested in site and year as random intercepts (Bates et al. 2015). To test the robustness of the LRR models, we performed leave‐one‐out sensitivity analyses by sequentially removing sites or years from the models (Figures S6 and S7). In a supplementary model, we further explored the three‐way interaction between event strength (|SPEI‐9|), event type, and treatment for the LRR of plot biomass, species richness, dominance, or evenness (Figure S8, Table S5).

Analyses were performed using R statistical software (R Core Team 2025). All linear models were evaluated with quartile‐quantile plots and residual plots using the ‘DHARMa’ (Hartig et al. 2024) and ‘performance’ packages (Ludecke et al. 2021). Pairwise comparisons were performed in the ‘emmeans’ package (Lenth et al. 2025) using a Tukey adjustment for multiple comparisons.

## 3 Results

**FIGURE 1:**
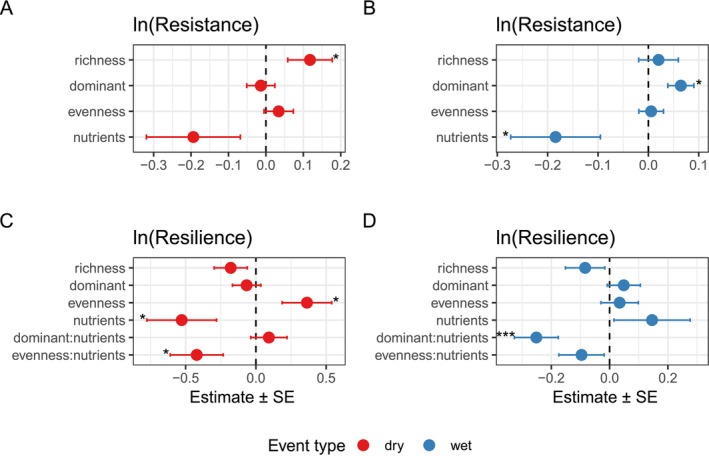
The effects of species richness, long‐term dominant species abundance, evenness and nutrient addition on aboveground plant biomass resistance and resilience to extreme dry and wet events. (A and B) show standardized regression coefficients for resistance, while (C and D) show standardized regression coefficients for resilience. Red and blue points represent responses to extreme dry and wet events, respectively. Error bars indicate standard errors of the coefficients. *p*‐values are denoted with asterisks (***< 0.0001, **< 0.001, *< 0.05).

Resistance and resilience to extreme climate events were predicted by species richness, evenness and dominance measured in the year prior to an extreme event (Figure 1, Table 1). Greater richness enhanced resistance to extreme dry events (estimate ± SE hereinafter unless otherwise stated = 0.12 ± 0.06, *p* = 0.048, Figure 1A). Greater evenness promoted resilience in control plots (0.36 ± 0.18, *p* = 0.04), but interacted with nutrient additions to reduce resilience during dry events (−0.42 ± 0.19, *p* = 0.026, Figure 1C, Figure S9). In contrast, greater dominance increased resistance to extreme wet events (0.06 ± 0.03, *p* = 0.013, Figure 1B), with nutrient addition decreasing resistance overall (−0.18 ± 0.09, *p* = 0.039). However, resilience to extreme wet events was negatively affected by the interaction of dominance and nutrient addition (−0.25 ± 0.08, *p* < 0.001, Figure 1D, Figure S9), such that greater dominance in nutrient‐addition plots lowered resilience.

**TABLE 1:** Standardized coefficients of the biotic and abiotic predictors of resistance and resilience to extreme dry and wet events. Bolded 𝛃 represents a significant predictor. The sign before the coefficient indicates the direction of the relationship. Degrees of freedom were estimated using Satterthwaite's methods.

Predictors	Extreme dry		Extreme wet	
	Resistance	Resilience	Resistance	Resilience
Intercept	***β* = 1.67 ± 0.24** **df = 4.4** ***p* = 0.0017**	***β* =** 0.87 ± 1.04 df = 1.19 *p* = 0.54	***β* = 1.43 ± 0.11** **df = 17.4** ***p* < 0.0001**	***β* =** 0.126 ± 0.19 df = 2.69 *p* = 0.56
Richness	***β* = 0.12 ± 0.06** **df = 534.8** ***p* = 0.048**	***β* =** −0.18 ± 0.12 df = 173.4 *p* = 0.13	***β* =** 0.02 ± 0.04 df = 300.3 *p* = 0.61	***β* =** −0.08 ± 0.07 df = 252.4 *p* = 0.22
Dominance	***β* =** −0.01 ± 0.04 df = 683.7 *p* = 0.72	***β* =** −0.07 ± 0.1 df = 490 *p* = 0.51	***β* = 0.06 ± 0.03** **df = 2281.9** ***p* = 0.013**	***β* =** 0.05 ± 0.06 df = 1551.7 *p* = 0.39
Evenness	***β* =** 0.03 ± 0.04 df = 786.5 *p* = 0.38	***β* = 0.36 ± 0.18** **df = 330.2** ***p* = 0.04**	**β =** 0.006 ± 0.02 df = 1758.8 *p* = 0.82	**β =** 0.04 ± 0.06 df = 1307.9 *p* = 0.59
Nutrients	***β* =** −0.19 ± 0.13 df = 369.6 *p* = 0.12	***β* = −0.53 ± 0.25** **df = 59.9** ***p* = 0.037**	***β* = −0.184 ± 0.09** **df = 385.4** ***p* = 0.039**	***β* =** 0.15 ± 0.13 df = 156.9 *p* = 0.27
Dominance × Nutrients	N/A	***β* =** 0.09 ± 0.13 df = 566.7 *p* = 0.47	N/A	***β* = −0.25 ± 0.08** **df = 1811.8** ***p* = 0.00087**
Evenness × Nutrients	N/A	***β* = −0.42 ± 0.19** **df = 354.7** ***p* = 0.026**	N/A	***β* =** −0.09 ± 0.08 df = 1881.6 *p* = 0.22

**FIGURE 2:**
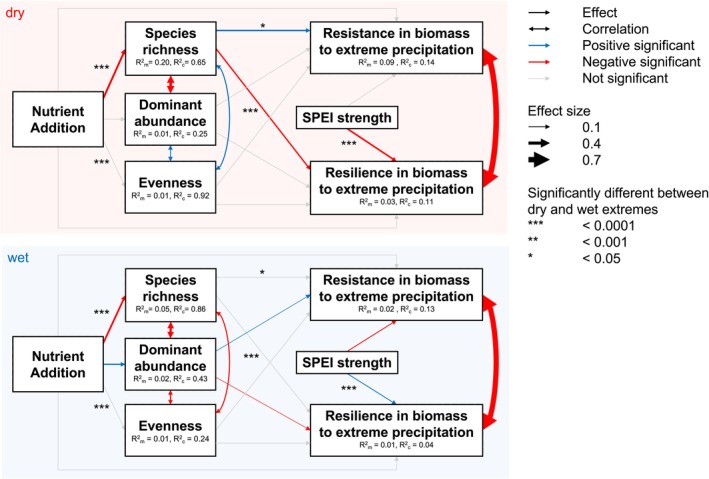
The direct and indirect effects of nutrient addition on aboveground plant biomass resistance and resilience to dry and wet extreme climate events. Shown is a multigroup structural equation model fit in the piecewiseSEM R package. Arrow size represents standardized model estimates. Dominant abundance is the abundance of long‐term dominant species.

The significant effects of nutrient addition on resistance and resilience were indirect and mediated through plant community properties (Figure 2, Tables S6 and S7). For extreme dry events, nutrient addition reduced species richness (standardized effect = −0.25, *p* < 0.001), with greater richness increasing resistance (standardized effect = 0.18, *p* = 0.014) and decreasing resilience (standardized effect = −0.18, *p* = 0.026). For extreme wet events, however, nutrient addition increased dominance (standardized effect = 0.15, *p* = 0.003) and decreased richness (standardized effect = −0.22, *p* < 0.0001); in turn, greater dominance increased resistance (standardized effect = 0.07, *p* = 0.014) but decreased resilience (standardized effect = −0.05, *p* = 0.042), with no richness effect on resistance and resilience. The strength of the extreme climate event also affected resistance and resilience; increasing dryness reduced resilience (standardized effect = −0.20, *p* < 0.001) whereas increasing wetness reduced resistance (standardized effect = −0.10, *p* < 0.001) yet increased resilience (standardized effect = 0.10, *p* < 0.001).

**FIGURE 3:**
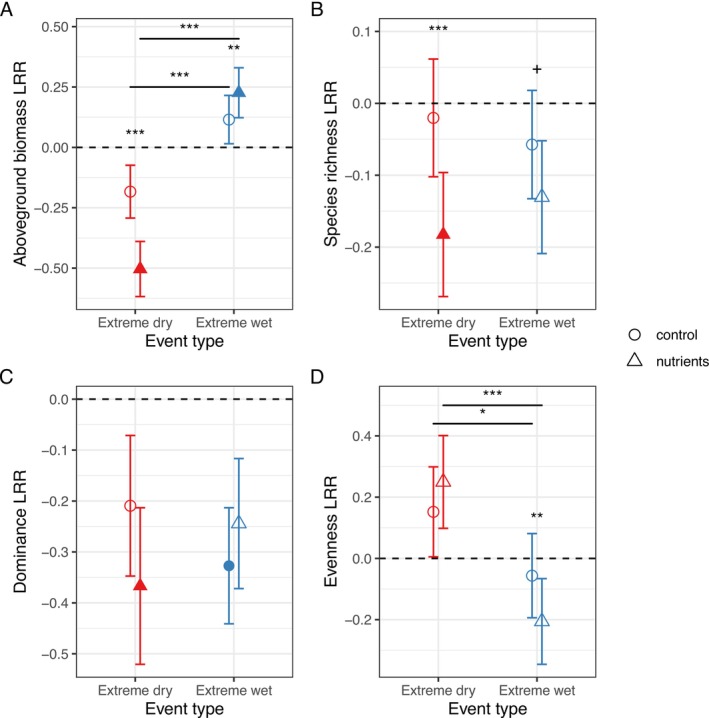
Log response ratio of (A) aboveground plant biomass, (B) species richness, (C) dominance (relative abundance of the long‐term dominant species) and (D) evenness to extreme dry and wet events. Points are predicted values (marginal effects). Error bars represent the standard error values. Points above zero (dashed line) show an increase in the response during the extreme events while points below zero show a decrease. Filled points are significantly different from zero (*p* < 0.05). *p*‐values are denoted with asterisks for comparisons between extreme dry events, between extreme wet events, between nutrient addition, and between control (***< 0.0001, **< 0.001, *< 0.05, +< 0.1).

During extreme climate events, nutrient addition generally magnified the effects of the event on species richness, dominance and aboveground biomass (Figure 3, Table S8). In control plots, plant biomass (−0.18 ± 0.11, *p* = 0.09), richness (−0.02 ± 0.08, *p* = 0.80) and dominance (−0.21 ± 0.14, *p* = 0.13) generally decreased, albeit insignificantly, during extreme dry events. However, nutrient addition significantly decreased biomass (−0.50 ± 0.11, *p* < 0.0001), richness (−0.18 ± 0.09, *p* = 0.03) and dominance (−0.37 ± 0.15, *p* = 0.02) during extreme dry events. In contrast, extreme wet events had less of an effect on plant community properties (Figure 3, Table S8). During extreme wet events, biomass increased in nutrient‐addition plots (0.23 ± 0.10, *p* = 0.029) and dominance decreased in control (−0.33 ± 0.11, *p* = 0.004) and nutrient‐addition plots (−0.24 ± 0.13, *p* = 0.056).

## 4 Discussion

By synthesizing up to four decades of data, we show that resistance and resilience of aboveground biomass to extreme climate events are determined by multiple plant community properties, including species richness, evenness and dominance. The influence of these community properties depends on the type of extreme event (dry vs. wet), while nutrient addition alters resistance and resilience indirectly via community properties. Our work builds on the foundational findings of Tilman and Downing (1994), which demonstrated that greater species richness stabilizes productivity during drought. While highly influential, that work has been debated (Aarssen 1997; Givnish 1994; Huston 1997; Huston et al. 2000) and refined over the past three decades (Chen et al. 2024; Isbell et al. 2015), with growing recognition that other components of plant community structure—particularly dominance (Hou et al. 2023; Smith et al. 2020) and evenness (Perez et al. 2024; Wang, Ge, et al. 2021; Wang, Isbell, et al. 2021; Yan et al. 2021)—determine ecosystem functioning and stability. Our findings support the richness–stability relationship in the case of drought, but they also reveal that dominant species play a stronger role in buffering responses to wet events and that evenness can enhance resilience under certain conditions. Furthermore, nutrient enrichment alters these dynamics indirectly by decreasing species richness and increasing dominance. Taken together, our results add ecological generality to long‐standing diversity–stability theory and partially resolve the debate around community drivers of stability by showing context‐dependent support for species richness, dominance and evenness, particularly under real‐world global change conditions.

The drivers of resistance varied depending on whether it was an extreme wet or dry event. Species richness was the most important community property that promoted resistance of aboveground biomass to extreme dry events (Figure 1A), which supports previous studies that experimentally manipulated species richness (Hossain et al. 2022; Isbell et al. 2015) and in naturally‐assembled communities (Tilman and Downing 1994). Under extreme dry conditions, communities tend to switch from competition to compensation as a greater number of species are more likely to include at least some species that can maintain biomass production (Bharath et al. 2020; Gonzalez and Loreau 2009; Loreau et al. 2021; Tilman and Downing 1994). Interestingly, we found a switch from species richness to dominant species abundance as the community property that predicted resistance to extreme wet events (Figure 1B). This is in contrast to previous studies which found species richness to be important for resistance to extreme wet events, but these studies did not examine the effects of dominant species (Hossain et al. 2022; Isbell et al. 2015). Our results may have shown this switch because dominant species, which drive biomass production, maintain rather than increase biomass in response to extreme wet events (Lisner et al. 2023; Gao et al. 2022). Additionally, it may require multiple consecutive extreme wet events for the replacement of a less responsive dominant species with a more responsive species to occur (Wilcox et al. 2016). Evenness did not promote resistance to extreme dry or wet events, in contrast to previous studies (Perez et al. 2024; Wang, Ge, et al. 2021), which may be because our study uses naturally‐assembled communities from multiple sites. Overall, while richness is often considered to increase resistance to extreme events, our results show that this effect is dependent on the type of extreme event and that dominant species could play a more important role in extreme wet events.

None of the community properties that predicted resistance to extreme events also predicted resilience. Instead, we found that evenness was a predictor for resilience in control plots to extreme dry events (Figure 1C). Evenness has been shown to increase asynchrony, facilitating a balanced portfolio of plant traits that can help communities rebound after extreme events (i.e., complementarity effect), highlighting the role multiple community properties play in shaping grassland response to extreme climate events (Oddershede et al. 2019; Wang, Isbell, et al. 2021; Yan et al. 2021). There was a non‐significant trend for richness to decrease resilience to extreme dry events (Figure 1C), which supports the findings of Hossain et al. (2022) but contradicts Isbell et al. (2015). None of our community properties increased resilience to extreme wet events (Figure 1D), suggesting that resilience to wet events may be independent of community properties. By accounting for the effects of multiple community properties, our results underpin the role of species richness in stabilizing productivity during specific types of extreme events, but also that other community properties may play a more key role in maintaining productivity under other types of extreme events.

These relationships were further complicated by nutrient addition. The effect of nutrient addition on resistance and resilience is difficult to understand, as fertilization may influence resistance and resilience directly or indirectly through plant community changes (Kiene et al. 2023) and it may also change the relative importance of different community properties for resistance and resilience. In our study, nutrient addition influenced both plant biomass resistance and resilience, but only significantly through changes in plant community properties (Figure 2). Similar to Bharath et al. (2020), we found nutrient addition reduced resistance but increased resilience to extreme dry events indirectly by reducing species richness (Figure 2). By contrast, in response to extreme wet events, nutrient addition increased resistance but decreased resilience indirectly by increasing dominance (see also Ma et al. 2023). Our SEM explained very little of the variance in resistance and resilience, suggesting that other factors such as other nutrients, other climate variables and local soil properties may be important to consider. Nevertheless, we show that nutrients shift the mechanism by which communities respond to extreme events, but losses in resistance or resilience are at least partially counterbalanced by gains of the other.

Not only did multiple community properties prior to the climate extreme events determine resistance and resilience to extreme climate events, but community properties during the climate extreme events were directly impacted by these events and were further modified by nutrient addition (Figure 3). In particular, nutrient addition amplified biomass responses, exacerbating losses during dry events and enhancing gains during wet events. This asymmetry aligns with previous findings (Van Sundert et al. 2021) and may result from nutrient‐induced decreases in root: shoot ratios that impair water uptake and retention during drought (Feng et al. 2023), or the alleviation of co‐limitation of water and nitrogen during wet events (Bondaruk et al. 2025; Ren et al. 2017). Although species richness remained relatively stable under climate extremes alone (Figure 3B; Hoover et al. 2014), it declined in fertilized plots during both extreme dry and wet events, suggesting that multiple global change factors in combination may be particularly detrimental to community diversity (Komatsu et al. 2019). Moreover, dominant species became less abundant during extreme events (Figure 3C), underscoring their context dependence and potentially limiting their stabilizing role under enriched and variable conditions. The reductions that extreme climate events cause in species richness and dominant species abundance, especially in nutrient‐enriched environments, may erode resistance and resilience to future events.

Our results contribute to the understanding of how plant communities respond to extreme climate events, which is essential for forecasting and managing ecosystems as the climate becomes less predictable and extreme events increase in frequency and intensity globally (IPCC 2023). Our study leveraged nearly four decades of data across three naturally‐assembled mesic grasslands to show that species richness, evenness and dominance influence ecosystem resistance and resilience—but that their roles depend on the type of extreme event and are further shaped by nutrient enrichment. Our multi‐site, long‐term approach enhances generality across North American tallgrass prairies and other mesic grassland ecosystems. Future research should examine consecutive extreme events and test whether these patterns extend to systems with different species pools, environmental constraints, or disturbance histories.

These findings collectively demonstrate that the stability response of these grassland communities to extreme climate events is context dependent. Specifically, the nature of the event (dry or wet) and nutrient enrichment determines which community properties will best predict resistance or resilience. In assessing the roles of species richness, evenness and dominance in driving ecosystem stability, our work shows that there is not one clear winner of the diversity‐stability debate for all global change scenarios. Instead, we must consider multiple components of biodiversity to cope with the dual pressures of nutrient pollution and intensifying climate variability.

## Author Contributions

All authors conceptualized the study and contributed to data compilation. Joshua A. Ajowele, Ashley L. Darst, Caitlin Broderick, Seraina L. Cappelli and Matthew A. Nieland: contributed to formal analyses. All authors contributed to writing and revising the manuscript.

## Funding

This work was supported by U.S. Forest Service, 20‐CS‐11091500‐009, 23‐CS‐11091500‐007. College of Natural Science, Michigan State University. National Institute of Food and Agriculture, 2020‐67019‐31171, 2024‐67012‐43175. Schweizerischer Nationalfonds zur Förderung der Wissenschaftlichen Forschung, P500PB_214352, P5R5–3_235083. Division of Environmental Biology, 0218210, 0620652, 0823341, 1234162, 1440484, 1831944, 1832042, 2025849, 2224712, 2425352, 9011662. Division of Integrative Organismal Systems, 9632851. National Science Foundation Graduate Research Fellowship Program, DGE: 184‐8739. Flory Cedar Creek Collaboration Fund. Minnesota Environment and Natural Resources Trust Fund.

## Supporting information


**Figure S1:** SPEI‐9 best explained the variation in aboveground plant biomass at our three sites. The red solid line represents the predicted values (marginal effects) with 95% confidence intervals. Each point is the aboveground biomass of one plot for 1 year. Note the *y*‐axis is displayed on the log_10_ scale.
**Figure S2:** SPEI‐9 values for Cedar Creek (CDR), Kellogg Biological Station (KBS), and Konza Prairie (KNZ) for all study years. Points above the blue dashed line were used as extreme wet years. Points below the red dashed line were used as extreme dry years.
**Figure S3:** Standardized coefficients of the biotic and abiotic predictors of resistance (A) and resilience (B) to extreme events (combined wet and dry extreme events). Error bars represent the standard error of the regression coefficients (***p = 0–0.001, **p < 0.001 = 0.01, *p > 0.01 < 0.05).
**Figure S4:** Leave‐one‐out sensitivity analyses to determine if a site strongly biased the biotic and abiotic predictors of resistance and resilience. No major bias was detected.
**Figure S5:** Leave‐one‐out sensitivity analyses to determine if the year an extreme event occurred strongly biased the biotic and abiotic predictors of resistance and resilience. No major bias was detected.
**Figure S6:** Leave‐one‐out sensitivity analyses to determine if a site strongly biased the log response ratios of aboveground biomass, species richness, dominance or evenness to extreme dry and wet years. No major bias was detected.
**Figure S7:** Leave‐one‐out sensitivity analyses to determine if the year an extreme event occurred strongly biased the log response ratios of aboveground biomass, species richness, dominance, or evenness to extreme dry and wet years. No major bias was detected.
**Figure S8:** Log response ratio of (A) aboveground plant biomass, (B) species richness, (C) dominance and (D) evenness to extreme dry and wet years. Solid lines are predicted values (marginal effects). Each point is the response of one plot during one extreme event year. Points above zero (dashed line) show an increase in the response during the extreme year while points below zero show a decrease. Plots were created using the ‘sjPlot’ R package.
**Figure S9:** Relationship between (A) resilience to dry extreme events and standardized evenness under control and nutrient enrichment, (B) resilience to wet extreme events and standardized relative abundance of the dominant species under control and nutrient enrichment.
**Table S1:** Predictions for the effects of extreme climate event type (wet or dry) and plant community properties on resistance and resilience of aboveground biomass. The symbol + represents a predicted positive relationship, − represents a predicted negative relationship, ± represents mixed responses, = represents no relationship and ? represents an unknown relationship. We limited supporting references to those that used similar definitions of resistance and resilience.
**Table S2:** Sources for compiled datasets used in analyses.

## Data Availability

All data are available on EDI at https://doi.org/10.6073/pasta/433a9ec43bfca734604b8c874895c991 (Ajowele et al. 2026) and code is available on Zenodo at https://doi.org/10.5281/zenodo.17202949.
