# Supplementary material for: Multiple Community Properties Drive Ecosystem Resistance and Resilience to Extreme Climate Events Across Mesic Grasslands
Source: Ecol Lett. 2026 Apr 7;29(4):e70380. doi: 10.1111/ele.70380 (PMC13054640; doi:10.1111/ele.70380)
Supplement: Supplementary file 1 — Figure S1: SPEI‐9 best explained the variation in aboveground plant biomass at our three sites. The red solid line represents the predicted values (marginal effects) with 95% confidence intervals. Each point is the aboveground biomass of one plot for 1 year. Note the y‐axis is displayed on the log10 scale. Figure S2: SPEI‐9 values for Cedar Creek (CDR), Kellogg Biological Station (KBS), and Konza Prairie (KNZ) for all study years. Points above the blue dashed line were used as extreme wet years. Points below the red dashed line were used as extreme dry years. Figure S3: Standardized coefficients of the biotic and abiotic predictors of resistance (A) and resilience (B) to extreme events (combined wet and dry extreme events). Error bars represent the standard error of the regression coefficients (***p = 0–0.001, **p < 0.001 = 0.01, *p > 0.01 < 0.05). Figure S4: Leave‐one‐out sensitivity analyses to determine if a site strongly biased the biotic and abiotic predictors of resistance and resilience. No major bias was detected. Figure S5: Leave‐one‐out sensitivity analyses to determine if the year an extreme event occurred strongly biased the biotic and abiotic predictors of resistance and resilience. No major bias was detected. Figure S6: Leave‐one‐out sensitivity analyses to determine if a site strongly biased the log response ratios of aboveground biomass, species richness, dominance or evenness to extreme dry and wet years. No major bias was detected. Figure S7: Leave‐one‐out sensitivity analyses to determine if the year an extreme event occurred strongly biased the log response ratios of aboveground biomass, species richness, dominance, or evenness to extreme dry and wet years. No major bias was detected. Figure S8: Log response ratio of (A) aboveground plant biomass, (B) species richness, (C) dominance and (D) evenness to extreme dry and wet years. Solid lines are predicted values (marginal effects). Each point is the response of one plot during one extreme event ye [file ELE-29-0-s001.docx]

**Supporting Information for** “Multiple community properties drive ecosystem resistance and resilience to extreme climate events across mesic grasslands”

**Authors:** Joshua A. Ajowele^*^, Ashley L. Darst^†^, Nameer R. Baker, Rachael R. Brenneman, Caitlin Broderick, Seraina L. Cappelli, Maowei Liang, Mary Linabury, Matthew A. Nieland, Maya Parker-Smith, Smriti Pehim Limbu, Rosalie S. Terry, Moriah Young, Max Zaret, Marissa Zaricor

^*^ Corresponding author: jaajowele@uncg.edu, joshuaajowele@gmail.com

^†^ Co-corresponding author: darstash000@gmail.com, darstash@msu.edu


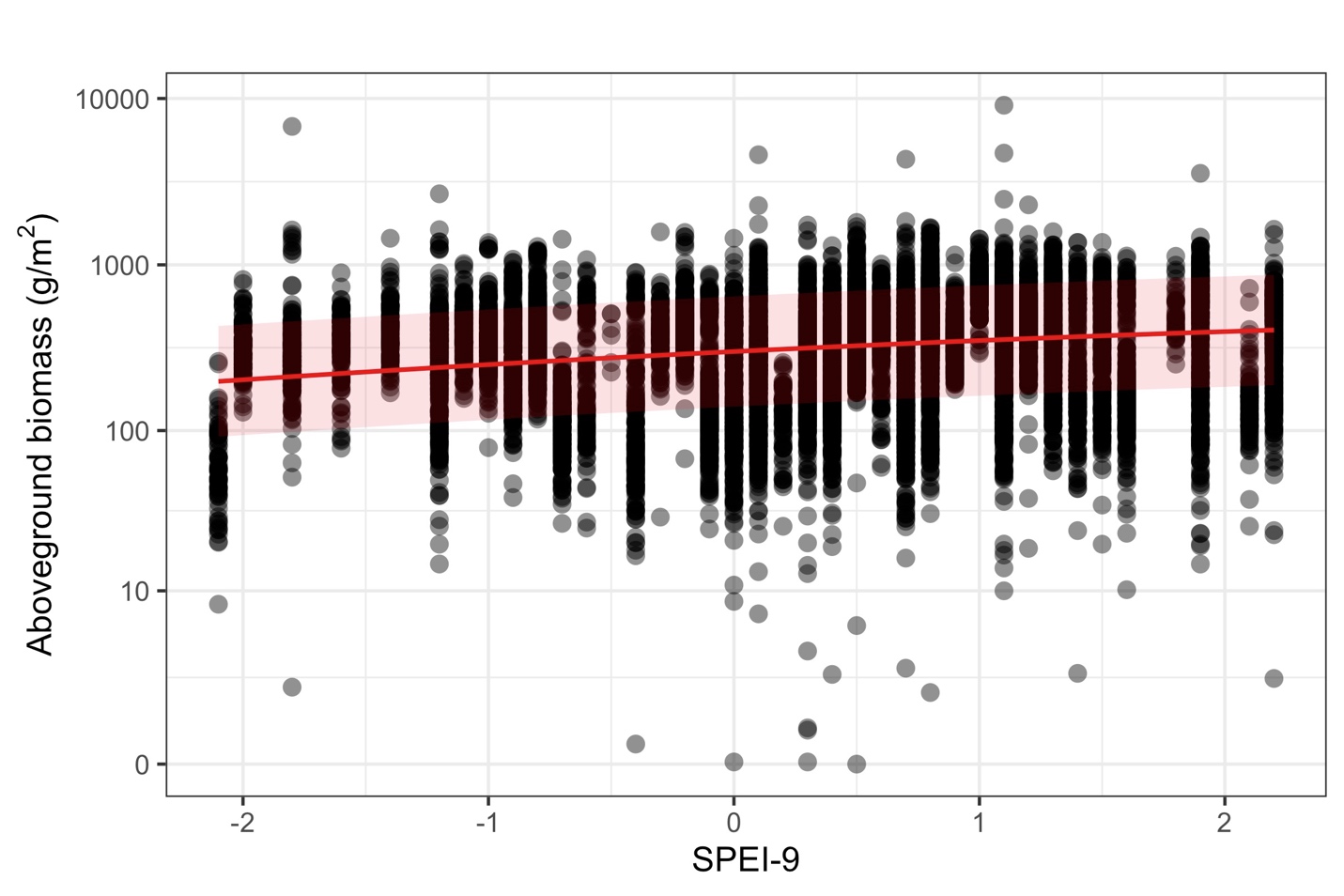


**Figure S1.** SPEI-9 best explained the variation in aboveground plant biomass at our three sites. The red solid line represents the predicted values (marginal effects) with 95% confidence intervals. Each point is the aboveground biomass of one plot for one year. Note the y-axis is displayed on the log_10_ scale.


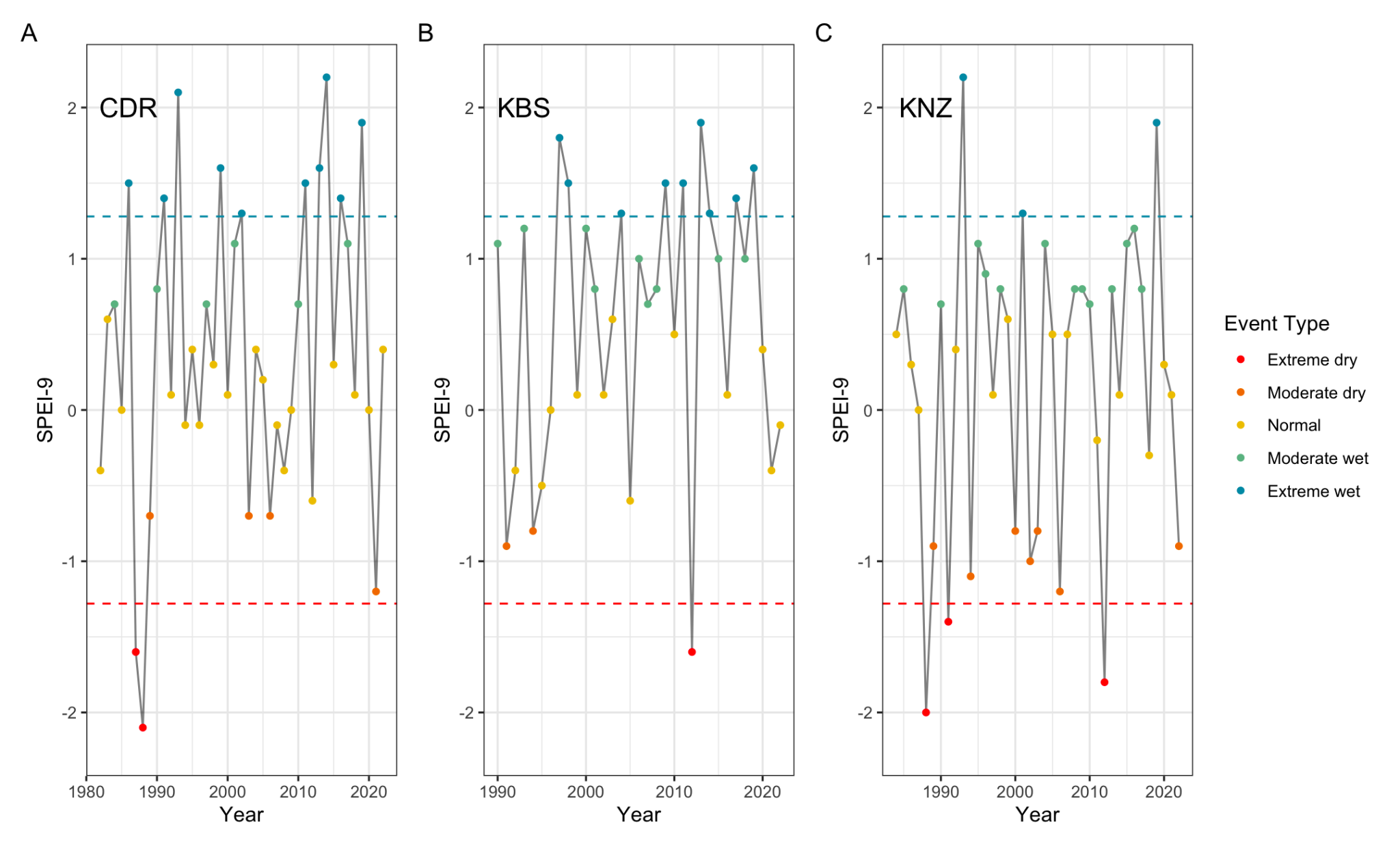


**Figure S2.** SPEI-9 values for Cedar Creek (CDR), Kellogg Biological Station (KBS), and Konza Prairie (KNZ) for all study years. Points above the blue dashed line were used as extreme wet years. Points below the red dashed line were used as extreme dry years.


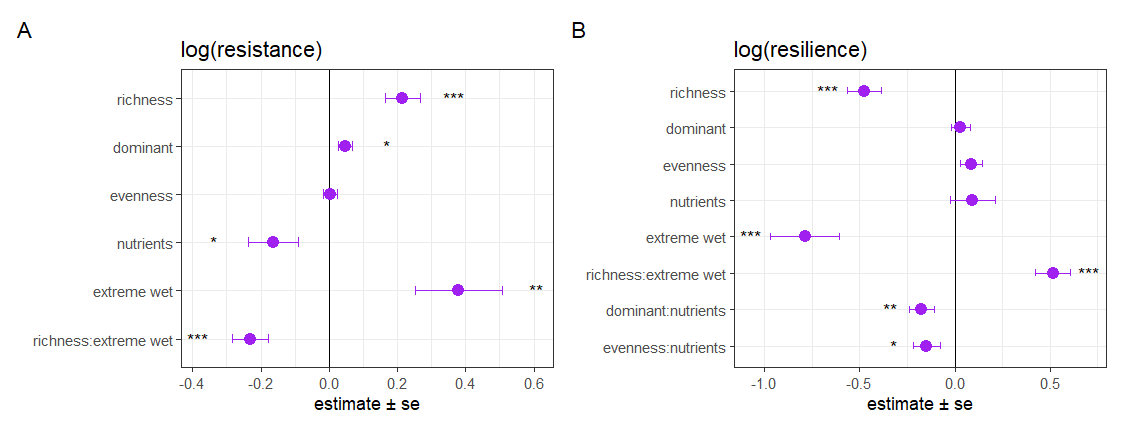


**Figure S3.** Standardized coefficients of the biotic and abiotic predictors of resistance (A) and resilience (B) to extreme events (combined wet and dry extreme events). Error bars represent the standard error of the regression coefficients (***p = 0-0.001, **p < 0.001=0.01, *p > 0.01<0.05).


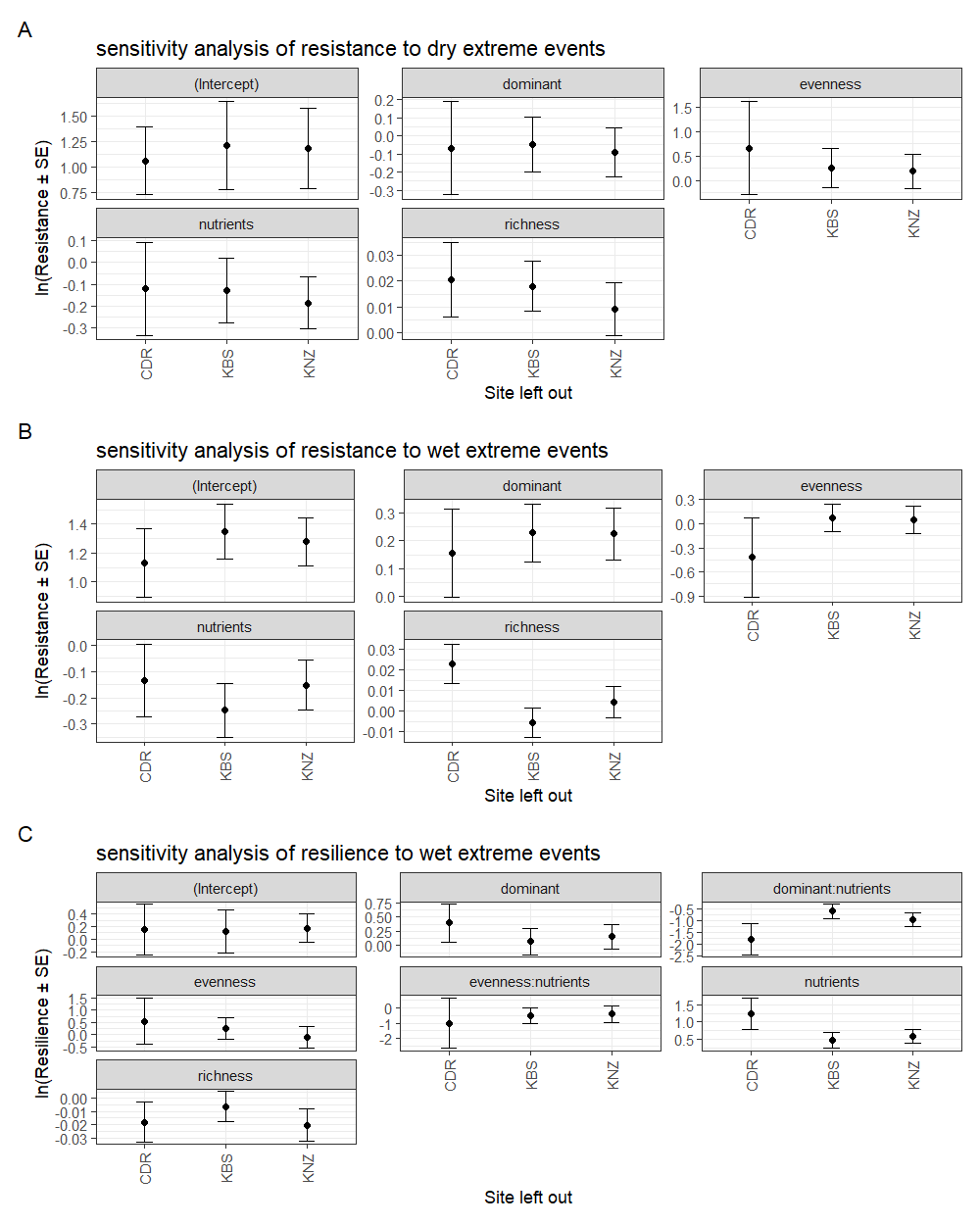


**Figure S4.** Leave-one-out sensitivity analyses to determine if a site strongly biased the biotic and abiotic predictors of resistance and resilience. No major bias was detected.


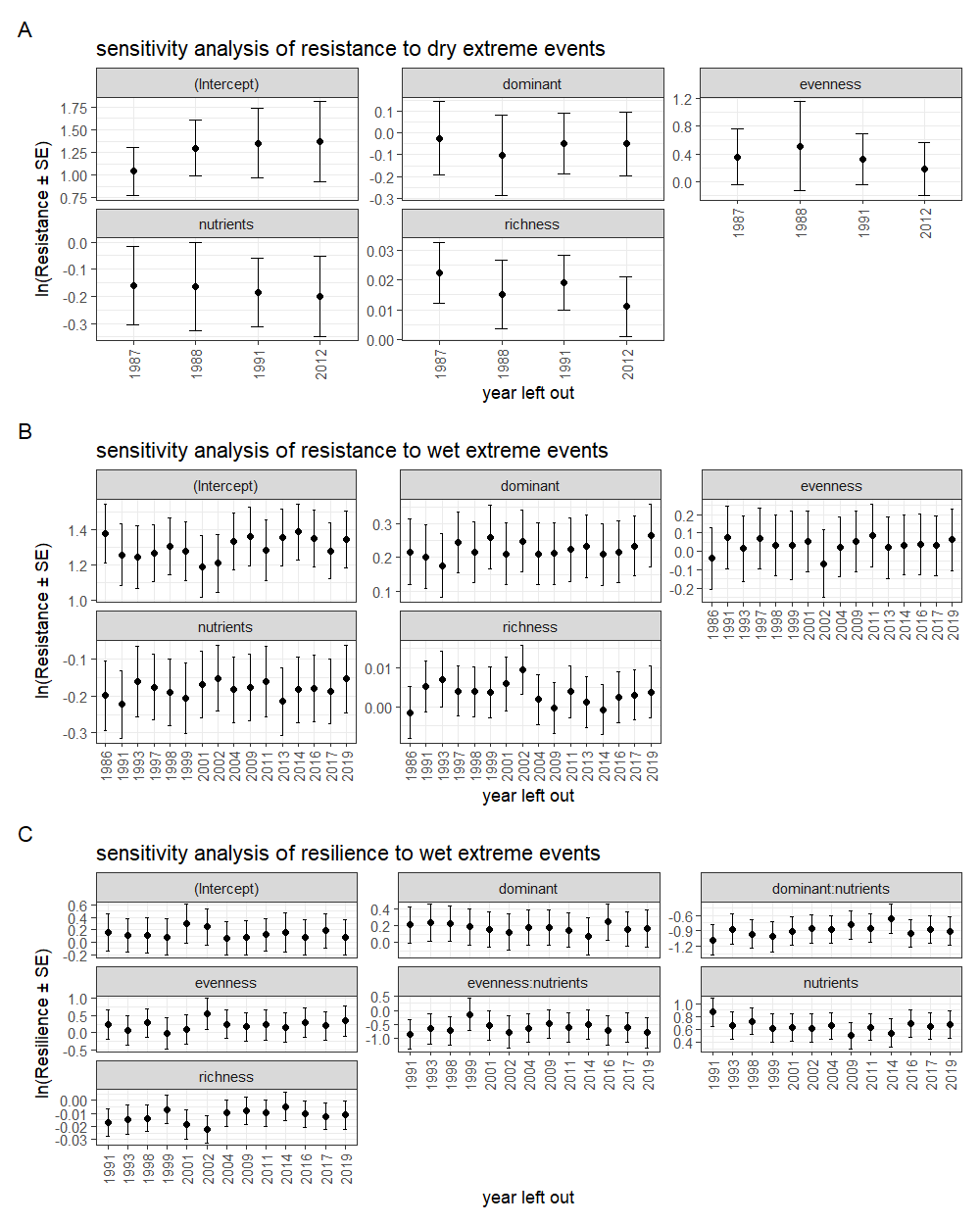
**Figure S5.** Leave-one-out sensitivity analyses to determine if the year an extreme event occurred strongly biased the biotic and abiotic predictors of resistance and resilience. No major bias was detected.


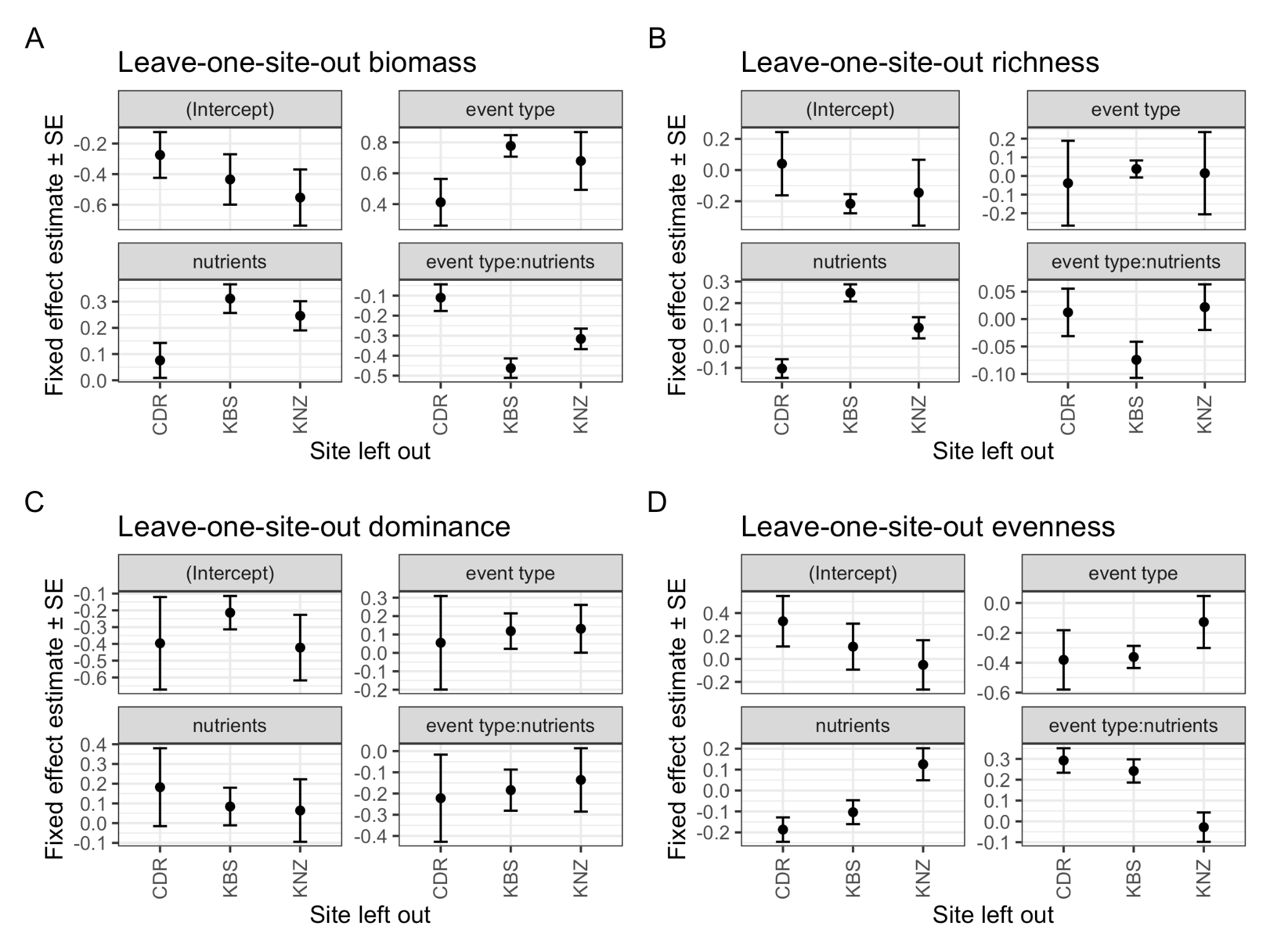
**Figure S6.** Leave-one-out sensitivity analyses to determine if a site strongly biased the log response ratios of aboveground biomass, species richness, dominance, or evenness to extreme dry and wet years. No major bias was detected.


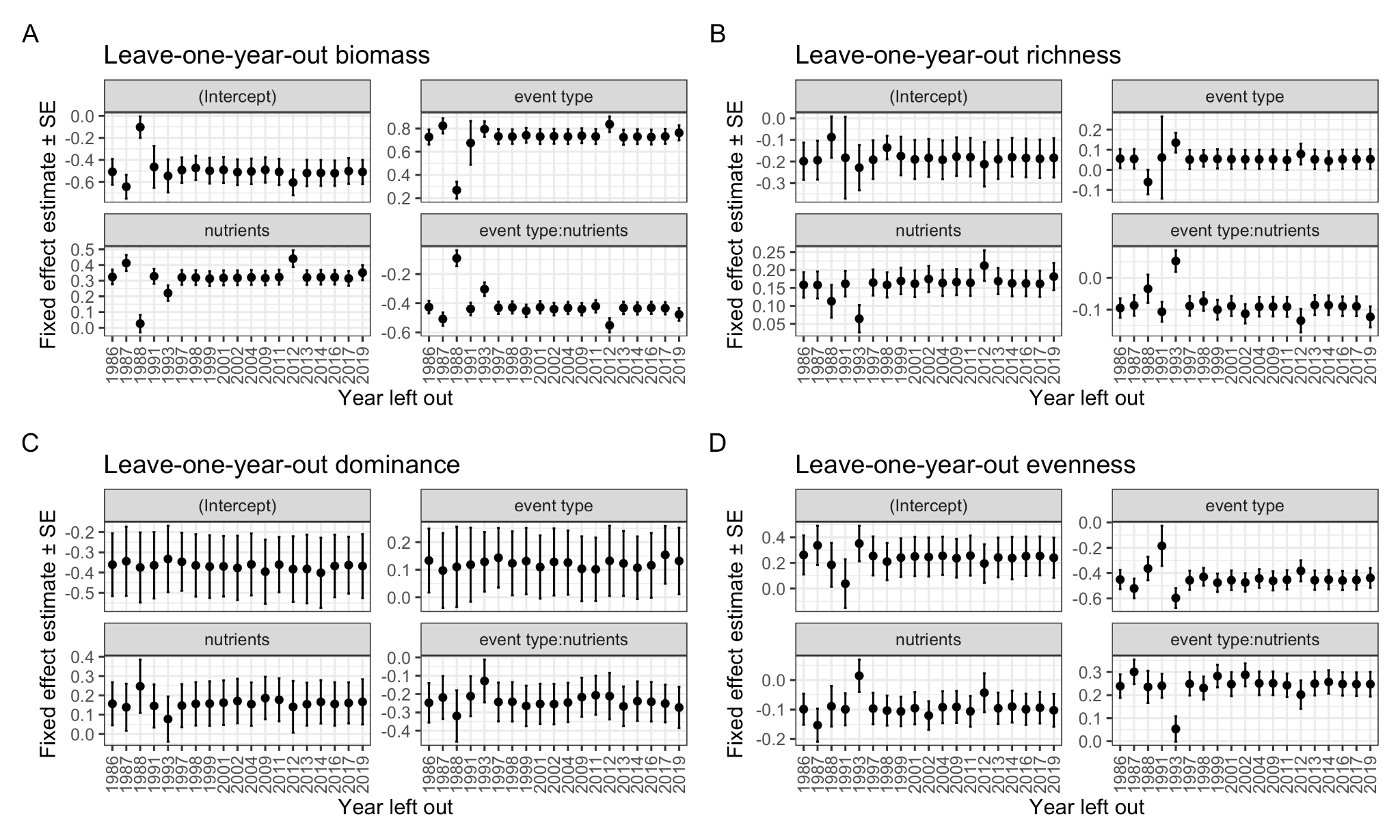


**Figure S7.** Leave-one-out sensitivity analyses to determine if the year an extreme event occurred strongly biased the log response ratios of aboveground biomass, species richness, dominance, or evenness to extreme dry and wet years. No major bias was detected.


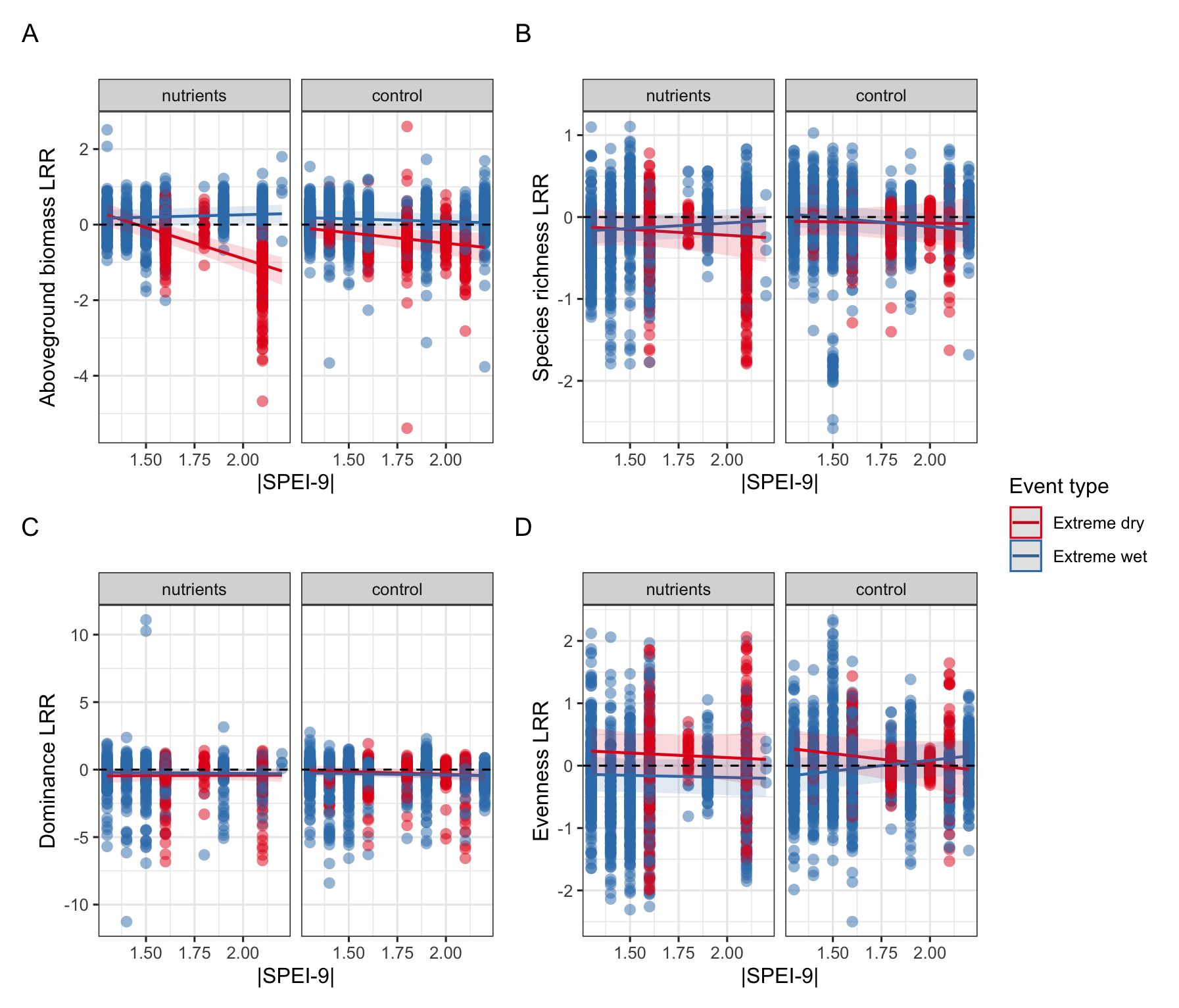


**Figure S8.** Log response ratio of A) aboveground plant biomass, B) species richness, C) dominance, and D) evenness to extreme dry and wet years. Solid lines are predicted values (marginal effects). Each point is the response of one plot during one extreme event year. Points above zero (dashed line) show an increase in the response during the extreme year while points below zero show a decrease. Plots were created using the ‘sjPlot’ R package.

**
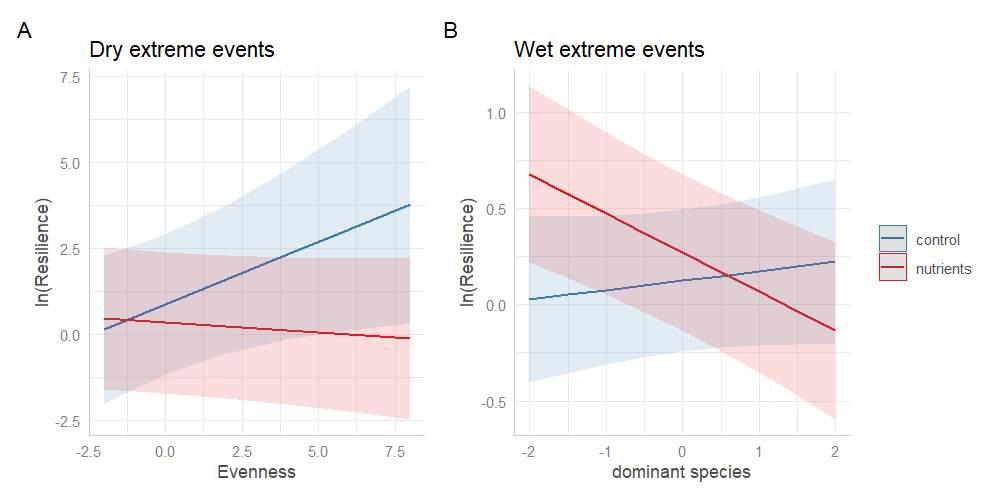
**

**Figure S9.** Relationship between A) resilience to dry extreme events and standardized evenness under control and nutrient enrichment, B) resilience to wet extreme events and standardized relative abundance of the dominant species under control and nutrient enrichment.

**Table S1. Predictions for the effects of extreme climate event type (wet or dry) and plant community properties on resistance and resilience of aboveground biomass.** The symbol + represents a predicted positive relationship, - represents a predicted negative relationship, +/- represents mixed responses, = represents no relationship, and ? represents an unknown relationship. We limited supporting references to those that used similar definitions of resistance and resilience.

| Response variable | Event type | Plant community variable | Response variable outcome | Reference |
| --- | --- | --- | --- | --- |
| Resistance | Wet | Species richness | +/- | Isbell et al. 2015; Hossain et al. 2022; Ma et al. 2023 |
|  |  | Dominance | + | Smith et al. 2020 |
|  |  | Evenness | + | Perez et al. 2024 |
|  |  | Nutrient addition | = | Ma et al. 2023 |
|  | Dry | Species richness | + | Isbell et al. 2015; Tilman & Downing 1994; Perez et al. 2024; Ma et al. 2023 |
|  |  | Dominance | + | Perez et al. 2024; Ma et al. 2023 |
|  |  | Evenness | + | Perez et al. 2024; Wang et al. 2021 |
|  |  | Nutrient addition | - | Bharath et al. 2020; Ma et al. 2023 |
| Resilience | Wet | Species richness | =/- | Isbell et al. 2015; Hossain et al. 2022; Ma et al. 2023 |
|  |  | Dominance | + | Ma et al. 2023 |
|  |  | Evenness | ? |  |
|  |  | Nutrient addition | +/- | Ma et al. 2023 |
|  | Dry | Species richness | +/- | Isbell et al. 2015; Hossain et al. 2022; Ma et al. 2023 |
|  |  | Dominance | + | Hoover et al. 2014; Ma et al. 2023 |
|  |  | Evenness | ? |  |
|  |  | Nutrient addition | +/- | Bharath et al. 2020; Ma et al. 2023 |

**Table S2. Sources for compiled datasets used in analyses.**

| Site | Dataset | Area sampled | Year range | Total years | (EDI) Citation |
| --- | --- | --- | --- | --- | --- |
| Cedar Creek | Long Term N Addition undisturbed e001 | 4 x 4m | 1982 - 2022 | 38 | Tilman, D. 2024. Plant aboveground biomass data: Long-Term Nitrogen Deposition: Population, Community, and Ecosystem Consequences ver 11. Environmental Data Initiative. https://doi.org/10.6073/pasta/2eba7aac6b347d27a92208e03fd3f8ea |
|  | Long Term N Addition disturbed e002 | 4 x 4m | 1982 - 2022 | 38 | Tilman, D. 2024. Plant aboveground biomass data: Long-Term Nitrogen Deposition During Grassland Succession ver 12. Environmental Data Initiative. https://doi.org/10.6073/pasta/66724d71711b80d520fa33a690f962b2 |
|  | Oldfield Chronosequence e054 | 1 x 1.5m | 1988 - 2022 | 35 | Tilman, D. 2024. Plant aboveground biomass data: Old-Field Chronosequence: Plant Productivity ver 10. Environmental Data Initiative. https://doi.org/10.6073/pasta/02d38edbe0860ef0a0555ff3e495ca1a |
|  | Oldfield Enemy Removal e245 | 3 x 3m  (after 2019 split into two 3m x 1.5m plots) | 2009 - 2021 | 13 | Kinkel, L. 2024. Plant aboveground biomass data: The influence of natural enemies on plant community composition and productivity ver 11. Environmental Data Initiative. https://doi.org/10.6073/pasta/303607d5f92929a4b20ba127c47d21f0 |
|  | Nutrient Network e247 |  | 2007 - 2018 | 12 | Seabloom, E. 2021. Aboveground Standing Crop Biomass:Nutrient Network A cross-site investigation of bottom-up control over herbaceous plant community dynamics and ecosystem function ver 9. Environmental Data Initiative. https://doi.org/10.6073/pasta/7c815175fc378270b6848351e43275b9  Seabloom, E. 2021. Plant Species Composition percent cover:Nutrient Network: A cross-site investigation of bottom-up control over herbaceous plant community dynamics and ecosystem function. ver 9. Environmental Data Initiative. https://doi.org/10.6073/pasta/da6f0efe58725ebddc4d5b59dd35bc8b |
| Kellogg Biological Station | Main cropping system experiment: early successional plots (T7) | 0.5 × 2.0 m | 1991 - 2022 | 32 | Robertson, G. and S. Snapp. 2020. Annual Net Primary Production on the Main Cropping System Experiment at the Kellogg Biological Station, Hickory Corners, MI (1990 to 2018) ver 85. Environmental Data Initiative. https://doi.org/10.6073/pasta/f9b2a038b8a7ad43a252587a84935f30 |
|  | Main cropping system experiment: early successional microplots (T7) | 0.5 × 2.0 m | 1990 - 2022 | 33 | Gross, K. and J. Lau. 2022. Plant Community and Ecosystem Responses to Long-term Fertilization & Disturbance at the Kellogg Biological Station, Hickory Corners, MI (1989 to 2019) ver 49. Environmental Data Initiative. https://doi.org/10.6073/pasta/ea22c735ddfe17c595ccca978a87d109 |
|  | GLBRC Biofuel Cropping System Experiment: early successional and restored prairie plots (G9 & G10) | 0.5 × 2.0 m | 2009 - 2017 | 9 | Not publicly available |
|  | GLBRC Scale-up: restored prairie plots (L3 & M2) | 0.5 × 2.0 m | 2010 - 2016 | 7 | Not publicly available |
|  | Nutrient network | 0.2 m^2^ (ANPP)  1 m^2^ (species cover) | 2013 - 2019 | 7 | Not publicly available |
| Konza Prairie | ChANGE Chronic Addition of Nitrogen Gradient Experiment | 0.1m^2^ (ANPP)  1 m^2^ (species cover) | 2014 - 2023 | 9 | Partially publicly available |
|  | Nutrient Network | 0.1m^2^ (ANPP)  1 m^2^ (species cover) | 2007 - 2022 | 15 | Komatsu, K. and M. Smith. 2023. NUT01 Nutrient Network: Investigating the roles of nutrient availability and vertebrate herbivory on grassland structure and function at Konza Prairie ver 7. Environmental Data Initiative. https://doi.org/10.6073/pasta/6e56960bb43fbcb29859c9e7178b7881. |
|  | Vertebrates and Invertebrates | 0.1m^2^ (ANPP)  1 m^2^ (species cover) | 2009 - 2022 | 13 | Komatsu, K., M. Smith, and A. Joern. 2023. VIR01 Effects of invertebate and vertebrate herbivory on tallgrass prairie plant community composition and biomass, Konza Prairie LTER ver 7. Environmental Data Initiative. https://doi.org/10.6073/pasta/5f70e62694b1c95efc25385d01a2521c. |
|  | Phosphorous Plots | 0.1m^2^ (ANPP)  1 m^2^ (species cover) | 2002 - 2021 | 19 | Avolio, M., K. Komatsu, and K. Wilcox. 2023. PPL01 Konza prairie long-term phosphorus plots experiment ver 5. Environmental Data Initiative. https://doi.org/10.6073/pasta/4dd3761c45fcb9a05f1df600f20b76b7. |
|  | Irrigation Transect Study |  | 1991 - 2021 | 30 | Blair, J. 2023. WAT01 Konza Prairie long-term irrigation transect study ver 22. Environmental Data Initiative. https://doi.org/10.6073/pasta/3037d44d496df3d12fbe9668c7d618a6. |
|  | Rainfall Manipulation |  | 1997 - 2012 | 15 | Smith, M., S. Collins, and J. Blair. 2023. RMP01 Rainfall manipulation plot study at Konza Prairie ver 3. Environmental Data Initiative. https://doi.org/10.6073/pasta/f8977cf7710e624e12f1a52e40cf883d. |
|  | Species Composition on Selected Watersheds | 10 m^2^ (species cover) | 1983 - 2022 | 39 | Hartnett, D., S. Collins, and Z. Ratajczak. 2023. PVC02 Plant species composition on selected watersheds at Konza Prairie ver 22. Environmental Data Initiative. https://doi.org/10.6073/pasta/0d591da0aff8bbcc8ec07c160d83d36e. |
|  | Aboveground Productivity Plots | 0.1m^2^ (ANPP) | 1983 - 2021 | 38 | Blair, J. and J. Nippert. 2024. PAB01 Aboveground net primary productivity of tallgrass prairie based on accumulated plant biomass on core LTER watersheds (001d, 004b, 020b) ver 17. Environmental Data Initiative. https://doi.org/10.6073/pasta/2a7ba5bc03267a29af74a188011041b0. |

**Table S3. Model comparison using different SPEI durations to predict aboveground biomass.** Aboveground biomass was transformed by natural logarithm. Plot nested in experiment nested in site and year were included as random intercepts. Models were fit using maximum likelihood to compare dAICc. The table is sorted by dAICc. The bolded model had the lowest dAICc and the highest marginal R^2^.

| Model terms | dAICc | R^2^_m_ | R^2^_c_ | df |
| --- | --- | --- | --- | --- |
| **SPEI-9 + SPEI-9^2^** | **0** | **0.0316** | **0.7699** | **8** |
| SPEI-9 | 3.0 | 0.0295 | 0.7688 | 7 |
| SPEI-12 | 113.5 | 0.0237 | 0.7655 | 7 |
| SPEI-12 + SPEI-12^2^ | 114.1 | 0.0243 | 0.7653 | 8 |
| SPEI-6 + SPEI-6^2^ | 114.7 | 0.0216 | 0.7624 | 8 |
| SPEI-6 | 116.0 | 0.0201 | 0.7613 | 7 |
| SPEI-3 + SPEI-3^2^ | 260.7 | 0.0101 | 0.7592 | 8 |
| SPEI-3 | 272.0 | 0.0071 | 0.7590 | 7 |

**Table S4. Standardized coefficient and standard error of the biotic and abiotic predictors of resistance and resilience to all extreme event types.** Bolded 𝛃 represents a significant predictor. The sign before the coefficient indicates the direction of the relationship.

| Predictors | All extreme event | |
| --- | --- | --- |
|  | Resistance | Resilience |
| Intercept | **𝛃 = 1.16 ± 0.15**  **df = 11.9**  **p < 0.0001** | **𝛃 = 0.77 ± 0.19**  **df = 29.1**  **p = 0.0003** |
| Richness | **𝛃 = 0.22 ± 0.05**  **df = 476.2**  **p < 0.0001** | **𝛃 = -0.48 ± 0.09**  **df = 563.7**  **p < 0.0001** |
| Dominance | **𝛃 = 0.05 ± 0.02**  **df = 2573.8**  **p = 0.029** | 𝛃 = 0.03 ± 0.05  df = 2007.7  p = 0.53 |
| Evenness | 𝛃 = 0.003 ± 0.02  df = 1695.1  p = 0.86 | 𝛃 = 0.09 ± 0.06  df = 1812.7  p = 0.13 |
| Event type (wet) | **𝛃 = 0.38 ± 0.13**  **df = 76.9**  **p = 0.0038** | **𝛃 = -0.79 ± 0.18**  **df = 40.7**  **p < 0.0001** |
| Nutrients | **𝛃 = -0.17 ± 0.07**  **df = 406.8**  **p = 0.025** | 𝛃 = 0.09 ± 0.12  df = 170.3  p = 0.43 |
| Richness x Event type (wet) | **𝛃 = -0.23 ± 0.05**  **df = 2357.7**  **p < 0.0001** | **𝛃 = 0.5 ± 0.09**  **df = 1236.7**  **p < 0.0001** |
| Dominance x Nutrients | N/A | **𝛃 = -0.18 ± 0.07**  **df = 2282.4**  **p = 0.0076** |
| Evenness x Nutrients | N/A | **𝛃 = -0.15 ± 0.07**  **df = 2367.7**  **p = 0.032** |

**Table S5. Selected contrasts of estimated marginal means of linear trends for predictors of plant community property changes during an event year.** Models were fitted with the log response ratio of aboveground plant biomass, species richness, dominance, or evenness as a function of the interaction between event strength, event type, and nutrients. Plot nested in experiment nested in site and year were included as random intercepts. Contrasts were performed using the ‘emtrends’ function in the ‘emmeans’ R package. P-values were adjusted using the Tukey method. Bolded values are significant at the alpha = 0.05 threshold.

| Model | Contrast | Marginal difference ± SE | P-value |
| --- | --- | --- | --- |
| Aboveground biomass | **Extreme dry nutrients - Extreme dry control** | **-1.098 ± 0.1700** | **< 0.0001** |
|  | **Extreme wet nutrients - Extreme wet control** | **0.271 ± 0.0695** | **0.0006** |
|  | Extreme dry control - Extreme wet control | -0.407 ± 0.2230 | 0.2637 |
|  | **Extreme dry nutrients - Extreme wet nutrients** | **-1.776 ± 0.2510** | **< 0.0001** |
| Species richness | Extreme dry nutrients - Extreme dry control | -0.1100 ± 0.1290 | 0.8275 |
|  | **Extreme wet nutrients - Extreme wet control** | **0.3419 ± 0.0511** | **< 0.0001** |
|  | Extreme dry control - Extreme wet control | 0.1788 ± 0.1910 | 0.7846 |
|  | Extreme dry nutrients - Extreme wet nutrients | -0.2731 ± 0.2010 | 0.5243 |
| Dominance | Extreme dry nutrients - Extreme dry control | 0.467 ± 0.399 | 0.6452 |
|  | Extreme wet nutrients - Extreme wet control | 0.086 ± 0.173 | 0.9598 |
|  | Extreme dry control - Extreme wet control | -0.225 ± 0.358 | 0.9228 |
|  | Extreme dry nutrients - Extreme wet nutrients | 0.156 ± 0.465 | 0.9871 |
| Evenness | Extreme dry nutrients - Extreme dry control | 0.2131 ± 0.2050 | 0.7258 |
|  | **Extreme wet nutrients - Extreme wet control** | **-0.4124 ± 0.0819** | **< 0.0001** |
|  | **Extreme dry control - Extreme wet control** | **-0.7063 ± 0.2630** | **0.0365** |
|  | Extreme dry nutrients - Extreme wet nutrients | -0.0808 ± 0.2950 | 0.9928 |

**Table S6. ​​Standardized and unstandardized effects in the multigroup SEM.** Significant effects (*P* < 0.05) are shown in bold and are highlighted with asterisks (p < 0.001 ***, p < 0.01 **, p < 0.05 *, p < 0.1 .). The SEM was created using ‘piecewiseSEM’. The model fit indices are X^2^ = 17.171 with p = 0.001 and Fishers’C = 44.674 and p = 0, which would indicate poor model fit. However, those modelfit indices are unreliable indicators of model fit for models fit on large data sets like ours, as they are sensitive to sample size.

|  | | **Extreme dry** | | | | | | **Extreme wet** | | | | | |
| --- | --- | --- | --- | --- | --- | --- | --- | --- | --- | --- | --- | --- | --- |
| **Response** | **Predictor** | **Est.** | **Std. Err.** | **Std. Est.** | **DF** | **P-value** |  | **Est.** | **Std. Err.** | **Std. Est.** | **DF** | **P-value** |  |
| Resistance | Event strength | -0.1288 | 0.0733 | -0.0942 | 502.53 | 0.079 | . | -0.1041 | 0.0244 | -0.1036 | 1385.1414 | **<0.001** | *** |
| Resistance | Richness | 0.1825 | 0.0734 | 0.1836 | 318.2002 | **0.014** | * | 0.0383 | 0.0472 | 0.0358 | 343.1074 | 0.418 |  |
| Resistance | Dominance | -0.0092 | 0.0483 | -0.0084 | 550.7109 | 0.849 |  | 0.0645 | 0.0262 | 0.0659 | 1628.9959 | **0.014** | * |
| Resistance | Evenness | 0.0468 | 0.0591 | 0.0337 | 539.2015 | 0.429 |  | -0.0008 | 0.0231 | -0.0008 | 1740.6606 | 0.974 |  |
| Resistance | Nutrient addition | -0.0787 | 0.156 | -0.0377 | 102.1412 | 0.615 |  | -0.0984 | 0.0944 | -0.047 | 510.1913 | 0.297 |  |
| Resilience | Event strength | -0.2893 | 0.0785 | -0.2031 | 529.8176 | **<0.001** | *** | 0.0985 | 0.0242 | 0.099 | 1312.3111 | **<0.001** | *** |
| Resilience | Richness | -0.1783 | 0.0795 | -0.1721 | 377.4042 | **0.025** | * | -0.0114 | 0.0412 | -0.0108 | 98.6457 | 0.782 |  |
| Resilience | Dominance | 0.005 | 0.0515 | 0.0043 | 550.8861 | 0.923 |  | -0.0526 | 0.0258 | -0.0543 | 1488.586 | **0.042** | * |
| Resilience | Evenness | -0.0232 | 0.0631 | -0.0161 | 546.0817 | 0.713 |  | -0.0105 | 0.0227 | -0.0113 | 1327.1717 | 0.643 |  |
| Resilience | Nutrient addition | -0.0096 | 0.1733 | -0.0044 | 128.3041 | 0.956 |  | 0.0573 | 0.086 | 0.0276 | 178.9713 | 0.506 |  |
| Richness | Nutrient addition | -0.5171 | 0.1058 | -0.2463 | 495.8275 | **<0.001** | *** | -0.4373 | 0.0658 | -0.2235 | 625.5509 | **<0.001** | *** |
| Dominance | Nutrient addition | 0.1386 | 0.1419 | 0.0727 | 72.1623 | 0.332 |  | 0.3313 | 0.1123 | 0.1546 | 548.6792 | **0.003** | ** |
| Evenness | Nutrient addition | 0.1542 | 0.1351 | 0.1024 | 137.3939 | 0.256 |  | 0.1685 | 0.0985 | 0.0756 | 655.1648 | 0.088 | . |

**Table S7. Model-wide interactions with event type (dry or wet) for the multigroup SEM.** Significant effects (*P* < 0.05) are shown in bold. The SEM was created using ‘piecewiseSEM’.

| Response | Predictor | P-value |
| --- | --- | --- |
| Resistance | Event type:Event strength | 0.9055 |
| **Resistance** | **Event type:Richness** | **0.0195** |
| Resistance | Event type:Dominance | 0.0988 |
| Resistance | Event type:Evenness | 0.1904 |
| Resistance | Event type:Nutrient addition | 0.8723 |
| **Resilience** | **Event type:Event strength** | **0.0002** |
| **Resilience** | **Event type:Richness** | **0.0002** |
| Resilience | Event type:Dominance | 0.3111 |
| Resilience | Event type:Evenness | 0.2516 |
| Resilience | Event type:Nutrient addition | 0.2324 |
| **Richness** | **Event type:Nutrient addition** | **< 0.0001** |
| Dominance | Event type:Nutrient addition | 0.1597 |
| **Evenness** | **Event type:Nutrient addition** | **0.0004** |

**Table S8. Estimated marginal means for predictors of plant community property changes during an event year.** Models were fitted with the log response ratio of aboveground plant biomass, species richness, dominance, or evenness as a function of the interaction between event type and nutrients using the ‘emmeans’ R package. Plot nested in experiment nested in site and year were included as random intercepts. P-values were adjusted using the Tukey method. Bolded values are significant at the alpha = 0.05 threshold.

| Model | Event type | Nutrients | Estimated marginal mean ± SE | P-value |
| --- | --- | --- | --- | --- |
| Aboveground biomass | Extreme dry | control | -0.183 ± 0.109 | 0.0939 |
|  | **Extreme dry** | **nutrients** | **-0.504 ± 0.114** | **< 0.0001** |
|  | Extreme wet | control | 0.115 ± 0.100 | 0.2503 |
|  | **Extreme wet** | **nutrients** | **0.226 ± 0.103** | **0.0286** |
| Species richness | Extreme dry | control | -0.0203 ± 0.0819 | 0.8045 |
|  | **Extreme dry** | **nutrients** | **-0.1825 ± 0.0863** | **0.0344** |
|  | Extreme wet | control | -0.0573 ± 0.0754 | 0.4469 |
|  | Extreme wet | nutrients | -0.1305 ± 0.0785 | 0.0963 |
| Dominance | Extreme dry | control | -0.209 ± 0.138 | 0.1293 |
|  | **Extreme dry** | **nutrients** | **-0.367 ± 0.154** | **0.0170** |
|  | **Extreme wet** | **control** | **-0.327 ± 0.114** | **0.0041** |
|  | Extreme wet | nutrients | -0.244 ± 0.128 | 0.0558 |
| Evenness | Extreme dry | control | 0.1521 ± 0.147 | 0.3004 |
|  | Extreme dry | nutrients | 0.2497 ± 0.151 | 0.0991 |
|  | Extreme wet | control | -0.0563 ± 0.137 | 0.6818 |
|  | Extreme wet | nutrients | -0.2059 ± 0.140 | 0.1410 |

**Equation S1.**

log(Response Variable)_i_​ = ​β_0_ ​+ β_R​(_richness_i​_) + β_D_​(dominance_i_​) + β_V_​(evenness_i​_) + β_Event_​(event type_i​_) + β_Trt_​(treatment_i_​) + β_R:Event_​(richness_i_​×event type_i_​) + β_R:Trt_​(richness_i_​×treatment_i_​) + β_D:Event_​(dominance_i_​×event type_i_​) + β_D:Trt_​(dominance_i_​×treatment_i_​) + β_V:Event_​(evenness_i_​×event type_i_​) + β_V:Trt_​(evenness_i_​×treatment_i​_) + β_Event:Trt_​(event type_i_​×treatment_i_​) + α_plot[i]_​ + α_experiment[i]​_ + α_site[i]​_ + α_year[i]_ ​+ ε_i​​_, where α_plot[i]​_ ∼ N(0, σ^2^_plot_), α_experiment[i]​_ ∼ N(0, σ^2^_experiment_​), α_site[i]_​ ∼ N(0, σ^2^_site_​), α_year[i]​_ ∼ N(0, σ^2^_year_​), ε_i​_ ∼N(0, σ^2^)

**Equation S2.**

log(Resistance_i_​) = ​β_0​_ + β_1_​(richness_i_​) + β_2_(dominance_i_​) + β_3_​(treatment)_i​_ + β_4_​(evenness_i​_) ​+ α_plot[i]_​ + α_experiment[i]​_ + α_site[i]​_ + α_year[i]_ ​+ ε_i​​_, where α_plot[i]​_ ∼ N(0, σ^2^_plot_), α_experiment[i]​_ ∼N(0, σ^2^_experiment_​), α_site[i]_​ ∼ N(0, σ^2^_site_​), α_year[i]​_ ∼ N(0, σ^2^_year_​), ε_i​_ ∼ N(0, σ^2^)

**Equation S3.**

log(Resilience_i_​) =​ β_0_ ​+ β_1_​(richness_i_​) + β_2_​(dominance_i_​) + β_3_​(treatment_i_​) + β_4_​(evenness_i_​) + β_5_(dominance_i_​×treatment_i_​) + β_6_​​(evenness_i_×treatment_i_​) + α_plot[i]​_ + α_experiment[i]​_ + α_site[i]​_ + α_year[i]​_ + ε_i​​_, where α_plot[i]​_ ∼ N(0, σ^2^_plot_), α_experiment[i]​_ ∼ N(0, σ^2^_experiment_​), α_site[i]_​ ∼N(0, σ^2^_site_​), α_year[i]​_ ∼ N(0, σ^2^_year_​), ε_i_​ ∼ N(0, σ^2^)
